# Arsenic

**Published:** 1878-07-20

**Authors:** 


### ARSENIC.

### RULES FOR ITS THERAPEUTIC USE.

Dr. L. D. Bulkley (New York *Medical Journal*, August, 1876), in an exhaustive article on the use and value of arsenic in the treatment of skin diseases, reaches the following conclusions:

1. Arsenic, when administered in medicinal doses, has quite another action from that manifested by poisonous doses. The average dose of the former is onetwenty-fourth of a grain of arsenious acid, while the smallest toxic dose is placed at two grains.

2. Arsenic, in medicinal doses, does not produce any slow poisoning, but has been administered for months or years in quantities, a small portion of whose aggregate amount would destroy life at once.

3. Arsenic, given by a careful practitioner, in doses to be effective, need never produce any symptoms which should cause regret.

4. Arsenic is eliminated very rapidly, chiefly by the bowels and kidneys, so that the urine shows evidences of it in a few hours. No trace of it can be found, on careful analysis of the body after death, two weeks after the last dose of arsenic. Arsenic, therefore, does not accumulate in the system.

5. The first symptom of a full dose of arsenic is usually a fullness about the face and eyes, and conjunctive irritation and tenderness. This need not be exceeded, but may often be kept up with advantage to a slight degree until the disease yields. Before any harm is done by arsenic, either this or a slight nausea or diarrhœa manifests itself.

6. Arsenic should always be given with or just after meals; it is often best to give it alone, or with a small amount of bitter infusion.

7. The bowels should be first well purged, and an occasional laxative will both assist the action of the drug and prevent or modify some of its unpleasant effects.

8. If the urine becomes loaded and the tongue coated, it is best to stop the medicine for a short time, and give diuretics. Some of these disturbances can be prevented by combining an alkali with the arsenic.

9. The most serviceable forms in which to use arsenic, named in the order of their value, are—solution of the chloride of arsenic, solution of the arseniate of potassa, that of the arseniate of soda, and the arseniate of ammonia, arsenious acid, iodide of arsenic, and the arseniates of iron and quinia.

10. The dose of arsenic, small at first, is to be increased slowly until some of its physiological effects are manifested or the disease yields; it may then be somewhat diminished.

11. It is very important that arsenic be taken very regularly and persistently, and always under the supervision and frequent inspection of the physician.

12. Arsenic is valuable in chronic rheumatism; hence, is useful in arthritic eruptions. It is serviceable in certain neuroses, as chorea and neuralgia; therefore, in skin diseases, with neurotic elements; it possesses anti-malarial properties, and is, consequently, serviceable in diseases of the skin, showing periodic symptoms, as intermittent urticaria, etc.; likewise, in patients with other skin diseases, who have been exposed to miasmatic influences.

13. Arsenic is certainly valuable in psoriasis, eczema, pemphigus, acne, and lichen in proper cases, and when due regard is paid to the secretory organs, and to diet and other elements of general health, of less certain value in lupus, ichthyosis, sycosis, verruca, and cancerous diseases. It is absolutely useless or harmful in the syphilo-dermata, the animal and vegetable parasitic diseases, in elephantiasis græcorum and arabum, in purpura, true prurigo, herpes zoster, scleroderma, molluscum contagiosum and fibrosum, keloid, nevus, etc.

14. The only local application of arsenic, which is justifiable, is either one where the strength is so weak, and the extent of its use so small, that there is no danger from absorption, which may occur when not expected, or one of such a strength as to kill adjoining tissue at once, and so prevent absorption.

